# Membrane Vesicles of Group B Streptococcus Disrupt Feto-Maternal Barrier Leading to Preterm Birth

**DOI:** 10.1371/journal.ppat.1005816

**Published:** 2016-09-01

**Authors:** Manalee Vishnu Surve, Anjali Anil, Kshama Ganesh Kamath, Smita Bhutda, Lakshmi Kavitha Sthanam, Arpan Pradhan, Rohit Srivastava, Bhakti Basu, Suryendu Dutta, Shamik Sen, Deepak Modi, Anirban Banerjee

**Affiliations:** 1 Dept. of Biosciences and Bioengineering, Indian Institute of Technology-Bombay, Mumbai, India; 2 Molecular Biology Division, Bhabha Atomic Research Centre, Mumbai, India; 3 Dept. of Earth Sciences, Indian Institute of Technology-Bombay, Mumbai, India; 4 Molecular and Cellular Biology Laboratory, National Institute for Research in Reproductive Health (ICMR), Mumbai, India; University of California, San Francisco, UNITED STATES

## Abstract

Infection of the genitourinary tract with Group B *Streptococcus* (GBS), an opportunistic gram positive pathogen, is associated with premature rupture of amniotic membrane and preterm birth. In this work, we demonstrate that GBS produces membrane vesicles (MVs) in a serotype independent manner. These MVs are loaded with virulence factors including extracellular matrix degrading proteases and pore forming toxins. Mice chorio-decidual membranes challenged with MVs *ex vivo* resulted in extensive collagen degradation leading to loss of stiffness and mechanical weakening. MVs when instilled vaginally are capable of anterograde transport in mouse reproductive tract. Intra-amniotic injections of GBS MVs in mice led to upregulation of pro-inflammatory cytokines and inflammation mimicking features of chorio-amnionitis; it also led to apoptosis in the chorio-decidual tissue. Instillation of MVs in the amniotic sac also resulted in intrauterine fetal death and preterm delivery. Our findings suggest that GBS MVs can independently orchestrate events at the feto-maternal interface causing chorio-amnionitis and membrane damage leading to preterm birth or fetal death.

## Author Summary

Preterm birth is a major health concern globally as it is not only a leading cause of neonatal death, but also has long term consequences including defective brain development. Infection of vagina and cervix of pregnant women with the bacteria, Group B *Streptococcus* (GBS), causes chorio-amnionitis that significantly increases the probability of preterm births. We report that, GBS produces small extracellular membrane vesicles (MVs) which are toxic to both fetal and maternal cells. In animal studies, we found that the MVs disrupt the connective tissue of the fetal membrane reducing its mechanical strength which may cause premature rupture of amniotic sac. Further we show that even in absence of the bacteria, the MVs directly led to extensive inflammation in the mouse resulting in chorio-amnionitis, preterm births and still births. Collectively, our findings reveal how GBS while colonizing the lower genitourinary tract might orchestrate events at the fetal membrane leading to premature birth.

## Introduction

Preterm birth is the leading cause of neonatal mortality worldwide [1]. Globally, an estimated 13 million babies are born prematurely each year, out of which more than one million succumb to death [2]. In addition, being the leading cause of neonatal death, preterm birth also increases the risk of neonatal infections [3]. The survivors of preterm birth are also at increased risk of neurodevelopmental impairments, respiratory and gastrointestinal complications [4].

Amongst the various causes of preterm birth, intrauterine infections by various bacterial pathogens have been suggested to be one of the main reasons [5]. Group B *Streptococcus* (*Streptococcus agalactiae*, GBS) the β-hemolytic, gram-positive bacteria colonizes the genitourinary tract of almost 20–30% pregnant women [6–8] and is frequently associated with preterm births [9]. Epidemiological and clinical studies have shown that colonization of vagina and cervix with GBS significantly increases the probability of intra-amniotic infection, chorio-amnionitis, endometriosis, preterm premature rupture of amniotic membrane (PPROM) and preterm births [10–15]. It is postulated that ascending GBS infection from the vagina to the feto-maternal space decreases amniotic membrane integrity causing PPROM [16]. This effect is attributed to the hemolytic pigment of GBS which causes chorio-amnionitis *in vivo* [17].

While bacterial infections have been strongly associated with preterm births, it is not clear how preterm labor-related infections occur. Although ascending infections are postulated to be the main reason of preterm births, recent studies have suggested that intra-amniotic inflammation associated with spontaneous preterm labor occurs even in the absence of detectable microorganisms in the feto-maternal interface and amniotic fluid, a phenomenon, referred to as ‘sterile intra-amniotic inflammation’ [18]. Similar observations were made in an experimental model of rhesus monkeys where GBS was not detected in the amniotic fluid despite extensive inflammation [19]. These observations led us to postulate that the physical presence of the bacteria in the amniotic fluid and/or the chorio-decidua may not be necessary for intra-amniotic inflammation and preterm birth.

Interaction with the environment and other units of life forms an important cellular phenomenon and is mediated via the action of either cell surface associated or secreted molecules. The latter bypasses the need for physical presence of the cell at the site of interaction which often might not be possible due to limitations of size, distance, presence of hostile molecules etc. Prokaryotes have a wide variety of secretion system which includes the classical secretory (Sec) system, the TAT system, accessory Sec system and ABC transporters. Apart from these, outer membrane vesicles secreted by gram-negative bacteria have been proposed to be an ancillary secretory mechanism. These bilayered structures were found to be secreted almost ubiquitously by most, if not all gram negative bacteria wherein they perform a wide range of functions including quorum sensing [20], biofilm formation [21], nutrient acquisition, defense [22] and stress resistance [23]. Lately, extracellular membrane vesicles (MVs) are also reported to be produced by a number of gram positive bacteria. These include *Staphylococcus aureus* [24], *Bacillus anthracis* [25], *Clostridium perfringens* [26], *Bacillus subtilis* [27] and very recently in *Streptococcus pneumoniae* [28] and *Streptococcus suis* [29]. Loaded with toxins and other virulence factors [25], adhesins and immuno-modulatory substances [30], these MVs contribute to the survival, virulence and dissemination of the pathogens in the host. While GBS is not known to produce similar vesicular structures; based on the observations in other pathogenic organisms, we hypothesized that GBS may also produce MVs, which at the feto-maternal interface/or amniotic fluid cause tissue damage resulting in PPROM and/or preterm delivery.

In this report, we demonstrate for the first time that independent of the strains, GBS produces MVs. These MVs are capable of anterograde transport in mouse reproductive tract, have collagenase activity and reduce the stiffness of mouse chorio-decidual membrane *ex vivo*. *In vivo* injection of GBS MVs in mouse amniotic sacs causes chorio-amnionitis and inflammation resulting in premature delivery and fetal demise. Collectively, these findings provide a novel insight into how GBS can orchestrate events at the fetal membrane leading to premature birth.

## Results

### Detection and isolation of extracellular membrane vesicles produced by GBS

**Fig 1:**
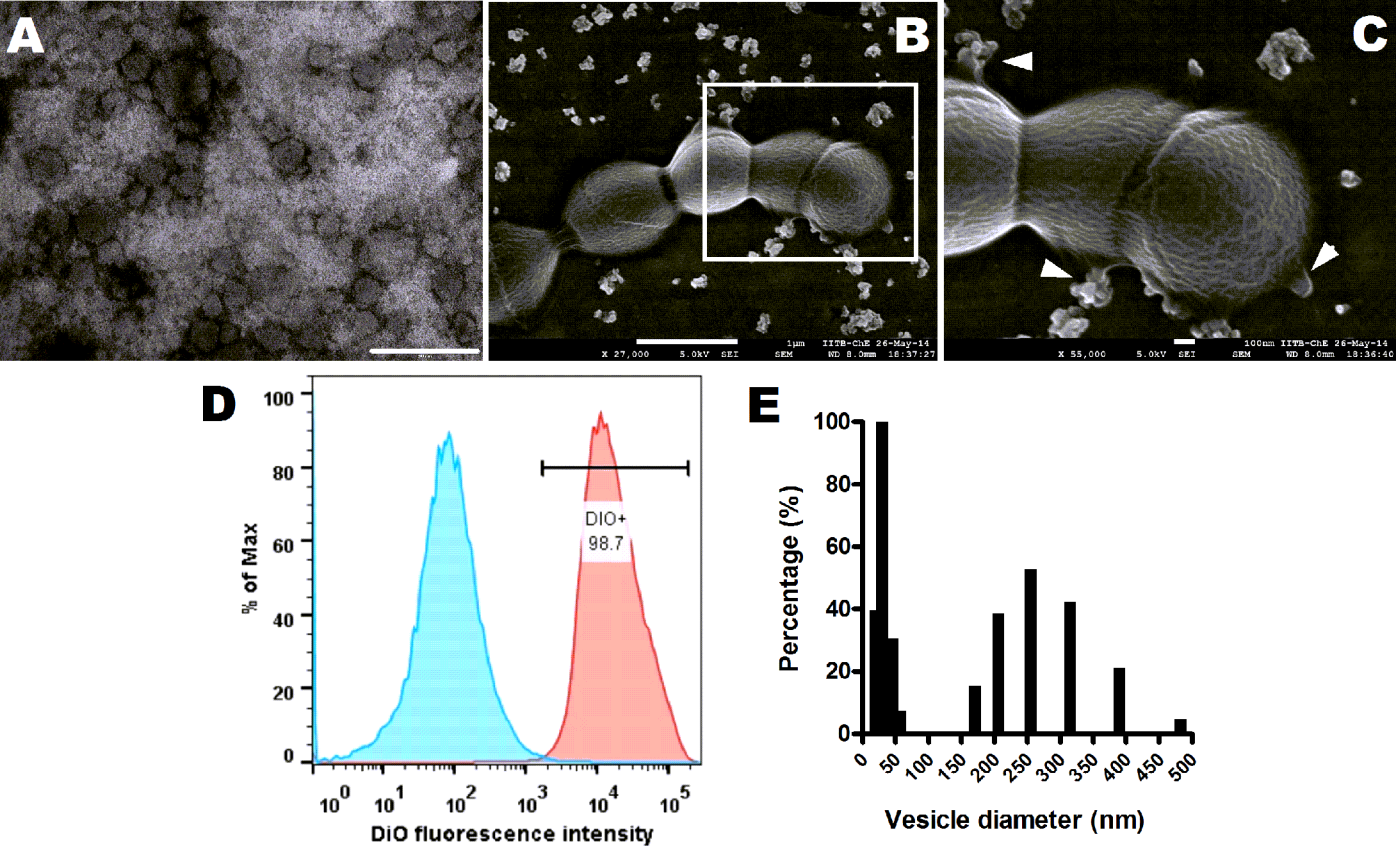
GBS produces extracellular membrane vesicles. **A.** Representative transmission electron micrograph of membrane vesicles (MVs) isolated from GBS strain A909. Scale bar; 200 nm. **B.** Representative scanning electron microscopic image of GBS strain A909 exhibiting aggregated MVs dispersed around the cells and budding of MVs from GBS cells. Scale bar; 1 μm. **C.** Zoomed in view of the boxed area in (**B**). Arrowheads indicate vesicles budding sites. Scale bar; 100 nm. **D.** Flow cytometry of isolated MVs stained with lipid specific dye, DiO. Blue shaded curve denotes unlabeled MVs and pink shaded curve depicts DiO labeled MVs. **E.** Size distribution of isolated MVs by Dynamic Light Scattering. Experiments were performed thrice and data from one representative experiment is shown.

Visualization of ultracentrifuged culture supernatant from GBS strain A909 (serotype IA) by Transmission Electron Microscopy (TEM) revealed numerous spherical structures of varying sizes resembling MVs (Fig 1A). The budding and release of MVs by GBS cells were observed by Scanning Electron Microscopy (SEM) where the vesicles were found to be aggregated both at the time of secretion and following discharge (Fig 1B and 1C). We also determined the presence of MVs associated with GBS by Atomic Force Microscopy (AFM) (S1A Fig). Along with A909, other GBS strains, including COH1 (serotype III), NEM316 (serotype III) and 2603 V/R (serotype V) were also found to secrete MVs (S1B Fig).

We stained the putative MVs from GBS strain A909 with DiO, a lipophilic fluorescent dye. Flow cytometry of the labeled MVs revealed that almost 99% of the vesicles were DiO positive (Fig 1D). Dynamic Light Scattering (DLS) of the vesicle preparations revealed two distinct populations, one < 50 nm and the other in the range of 150–300 nm (Fig 1E). Some larger structures (> 300 nm) observed in DLS analysis was thought to be vesicular aggregates, supporting our observations from SEM analysis. Quantitatively, 1 ml of late exponentially grown GBS culture was estimated to produce 1.7x10^4^ vesicles. The number of vesicles increased in a growth phase dependent manner, mostly due to increase in cell numbers. Maximum number of vesicles were attained at an OD of 1.2 corresponding to late-exponential phase of growth for GBS (S1C Fig).

### Characterization of MVs from GBS strain A909 and interaction with host cells

**Fig 2:**
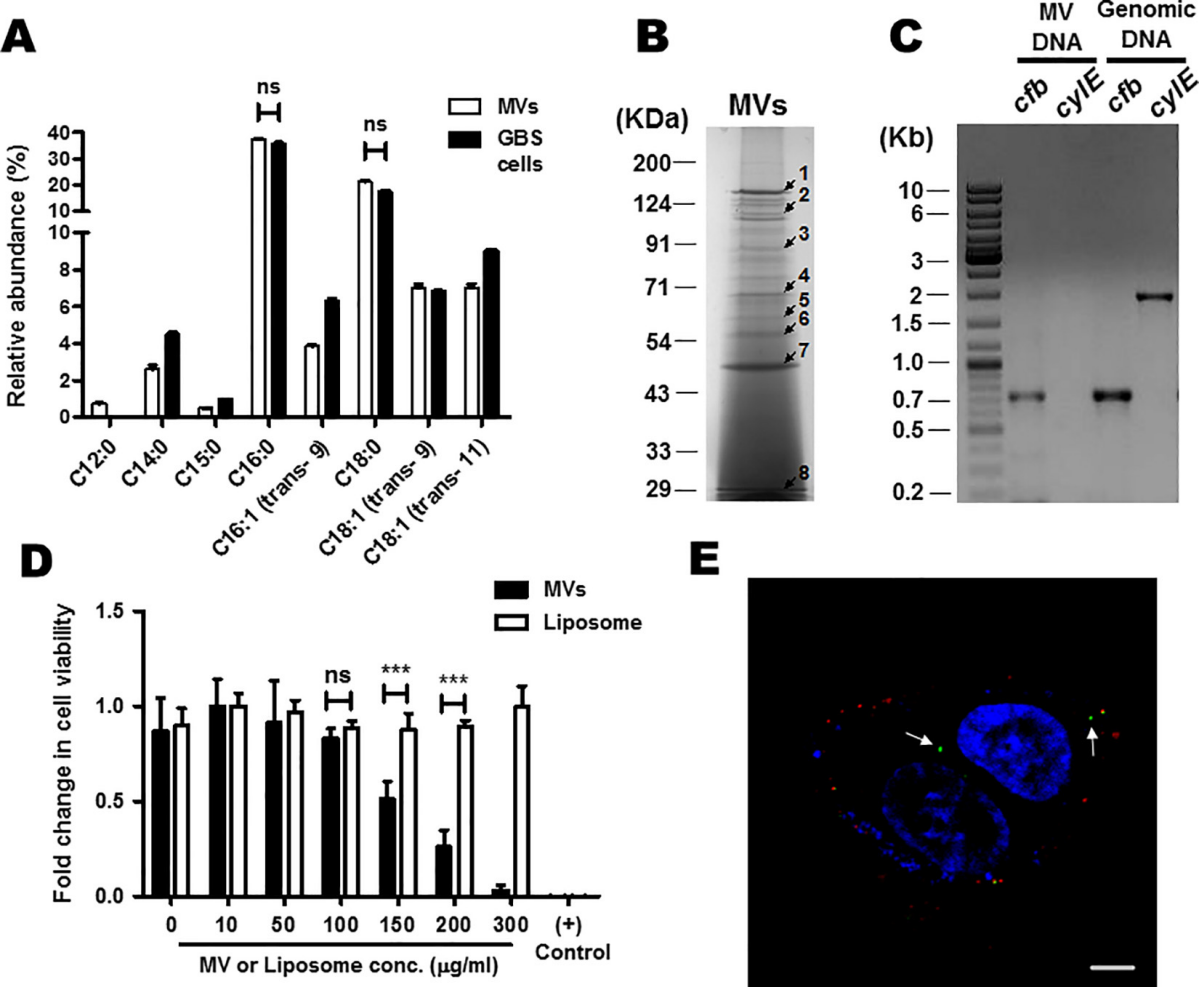
Characterization and biological activity of membrane vesicles isolated from GBS strain A909. **A.** Relative abundance of different fatty acids in MVs and whole bacterial cells analyzed using a GC-MS. Compounds were identified by comparison of the mass spectra with a library. Total fatty acids and other small molecules found in intact GBS cells were considered as 100% and the relative amounts of the same for MVs were calculated accordingly. Experiments were performed thrice. Statistical analysis was performed using two-way ANOVA (Bonferroni test); ns, non-significant. **B.** SDS-PAGE of proteins packaged in MVs. 150 μg of MV protein was precipitated with TCA (10%) and separated on 12% SDS-PAGE. Selected protein bands (indicated by arrow) were excised from the gel, trypsinized and the resulting peptides were analyzed by MALDI mass spectrometry followed by identification with Mascot search engine. The IDs of the corresponding proteins are given in Table 1. **C.** PCR amplification of *cylE* and *cfb* genes in DNA isolated from GBS cells or MVs. Lane 1: DNA ladder (1 Kb). The corresponding size of bands in Kb are marked. **D.** Cytotoxicity of MVs to HeLa cells. HeLa cells were incubated with different conc. of MVs or BSA entrapped liposomes (10 to 300 μg of protein/ml) for 24 h and cell viability was determined by MTT assay. 1% Triton X-100 solution was used as a positive control. Experiments were performed three times (each in triplicate) and results are expressed as fold change in cell viability with respect to highest value. Bars are mean ± standard deviation. Statistical analysis was performed using two-way ANOVA (Bonferroni test); ***p < 0.001; ns, non-significant. **E.** Internalization of GBS MVs in HeLa cells. FITC labeled MVs were incubated with HeLa cells for 6 h. Extracellular MVs were stained with anti-FITC Ab, followed by AlexaFluor 555 conjugated secondary antibody. Internalized MVs showed green fluorescence (white arrows) while the extracellular MVs demonstrated red fluorescence. Scale bar; 5 μm.

We characterized the MVs isolated from GBS strain A909 with respect to its composition. Comparison of the fatty acid profiles of the lipids isolated from MVs and GBS cells using GC-MS revealed that both intact bacterial cells and vesicles had similar lipid compositions with palmitic acid being the major fatty acid in both cases (Fig 2A).

We next analyzed the protein composition of GBS MVs by separating the total proteins in SDS-PAGE followed by peptide mass fingerprinting by MALDI mass spectrometry (Fig 2B). Eight out of 22 protein bands in a Coomassie stained SDS-PAGE gel used for GBS strain A909 MV proteins, could be successfully sequenced (Fig 2B). The list of identified proteins, their MASCOT scores and other details are provided in Table 1. *In silico* analysis revealed that all these proteins were either secreted or cell wall/membrane associated virulence factors (Table 1).

**Table 1:** GBS MV associated proteins and their predicted biological function.

Band No.^a^	Locus	Protein ID	Product	Mol. wt. (kDa)	Mascot Score	No. of peptides matched	Seq. coverage (%)	Location^b^	Biological role
1	SAK_0186	YP_328859.1	IgA binding beta antigen	131	215	39	36	Cell wall	Phagocytosis escape
2	SAK_1284	YP_329897.1	Hyaluronate lyase	122	208	37	37	Cell membrane	ECM degradation
3	SAK_1293	YP_329906.1	Neutral metallo protease fused to ChW-repeats	94	199	30	40	Cell membrane	ECM degradation
4	SAK_1695	YP_330297.1	Immunogenic secreted protein	55	122	17	48	Extracellular	Predicted hydrolase activity
5	SAK_0206	YP_328879.1	Oligopeptide ABC transporter oligopeptide binding protein	60	136	19	43	Extracellular	Transporter
6	SAK_0887	YP_329509.1	Elongation factor Tu	44	222	25	63	Cytoplasm	Translation elongation
7	SAK_0050	YP_328743.1	PcsB protein	45	93	9	43	Cell membrane	Cell division
8	SAK_1983	YP_330575.1	cAMP factor	28	76	10	40	Cell membrane	Pore forming toxin

^a^See Fig 2B.

^b^Subcellular locations were predicted by LocateP database and confirmed by pSORT and TMHMM algorithms.

The vesicles from strain A909 also contain DNA at a concentration of ~33 ± 5 ng/μg of MV protein. We attempted to amplify few genes encoding various GBS virulence factors, such as, *cylE* (acyl CoA acyl transferase involved in hemolysin synthesis), *cfb* (CAMP factor), *pepB* (metallopeptidase), *zooA* (zoocin) and *gapN* (glyceraldehyde 3-phosphate dehydrogenase) from the DNA isolated from MVs. A PCR product (750 bp) was obtained when primers specific for *cfb* gene was used (Fig 2C). No PCR products were detected when primer sets of other genes (*cylE*, *pepB*, *zooA* and *gapN*) were used for PCR, revealing the absence of the cognate DNA from isolated MVs. However, all these genes could be successfully amplified when genomic DNA of GBS was used as template in PCR.

We next attempted to determine if MVs can interact with host cells by studying the effects of purified MVs from GBS strain A909 on HeLa cells. A significant dose dependent reduction in the viability of HeLa cells was observed within 24 h of challenge with MVs. The reduction in viability was statistically significant at concentrations greater than 100 μg/ml (Fig 2D). BSA entrapped liposomes that were used as negative control had no effect on HeLa cells viability even at higher concentrations (Fig 2D), revealing that the cytotoxicity was specific for GBS MVs.

To determine if MVs can enter host cells, HeLa cells were incubated with fluorescently labeled MVs and visualized using a confocal microscope. FITC labeled MVs could be readily detected inside HeLa cells (Fig 2E). Extracellular MVs could be distinguished from the internalized MVs by double immunofluorescence where the internalized vesicles remain green and the extracellular MVs are red (Fig 2E).

### GBS MVs have collagenase activity and lead to mechanical weakening of chorio-decidual membranes

**Fig 3:**
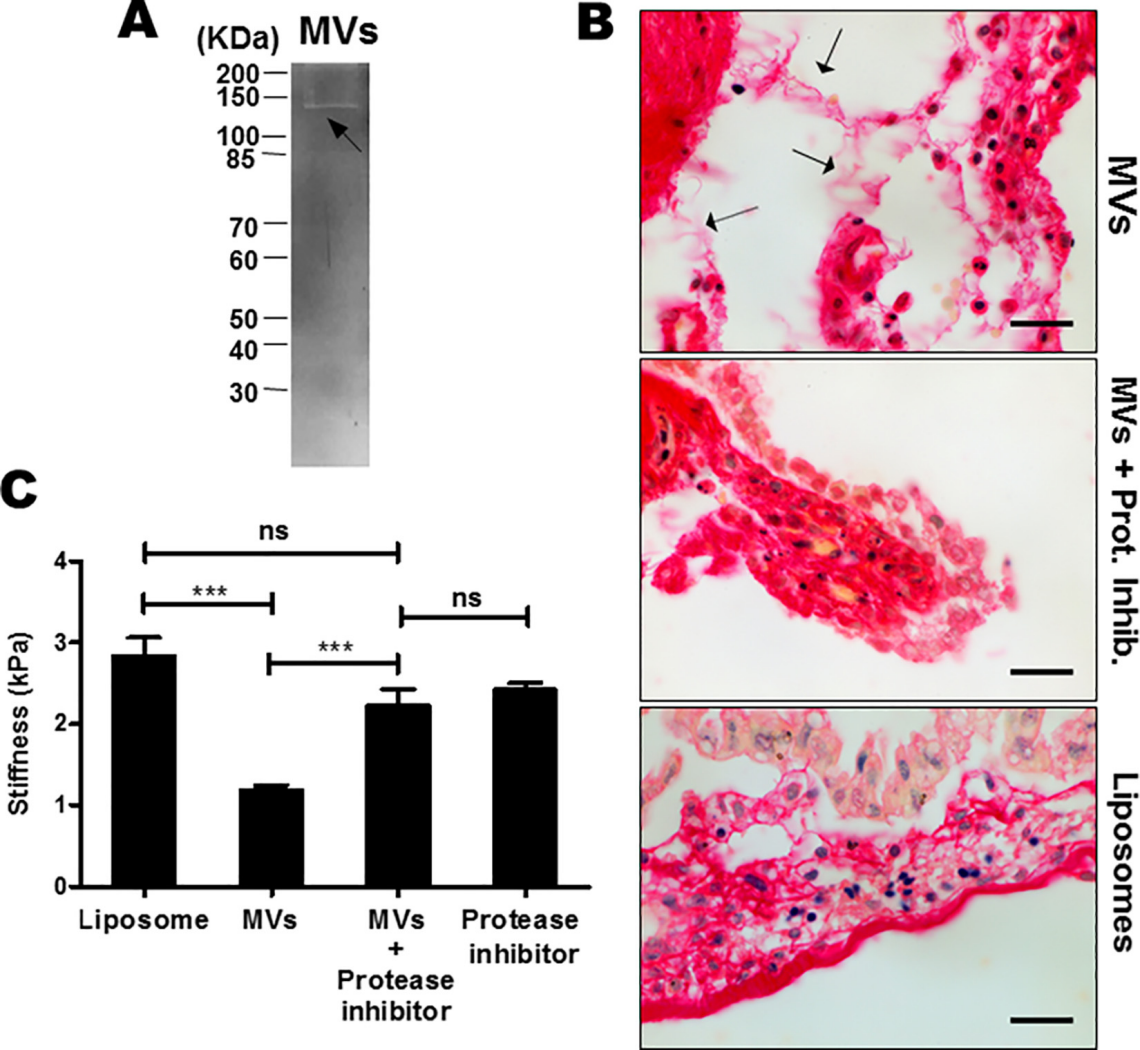
Collagenase activity of GBS MVs results in mechanical weakening of mouse chorio-decidual membrane *ex vivo*. **A.** Gelatin zymography of GBS MVs was performed using 10 μg of protein on 10% SDS-PAGE and stained with Coomassie Brilliant blue. Clear band (arrow) indicates the gelatinolytic activity of the MV protein. **B.** Effect of GBS MVs on collagen in the mouse chorio-decidual tissue. Chorio-decidual membrane on E14.5 were exposed to MVs, liposomes and protease inhibitors (PI) with and without MVs for 24 h and stained for total collagen using Picrosirius red. Collagen was detected as intense pink to red staining, blue/black stain is nuclei. Arrows indicate collagen fiber disintegration in MV injected tissues. Scale bar; 200 μm. **C.** Atomic Force Microscopy to estimate the effects of MVs on stiffness of the chorio-decidua. Mouse chorio-decidua (at E14.5) were incubated with MVs or liposomes or protease inhibitor cocktail (PI) with or without MVs for 24 h and probed at multiple positions over a randomly selected region of 50 μm X 50 μm. Force-indentation curves were fitted with Hertz model to estimate the Young’s modulus of elasticity. Experiments were performed thrice and bars represent standard deviation of the mean of one representative experiment. Statistical analysis was performed using one-way ANOVA (Tukey’s multiple comparison test); ***p < 0.001; ns, non-significant.

Since GBS MVs contain few ECM degrading proteases (Table 1), we tested the presence of collagenolytic activity in MVs from GBS strain A909 by gelatin zymography which resulted in a single clear band (Fig 3A). This underlines the presence of gelatinolytic activity in GBS MVs.

To test if the GBS MVs degrade collagen in the host tissues, mouse chorio-decidual membranes were incubated with MVs from strain A909 and stained for collagen (Fig 3B). In liposome (BSA entrapped) treated membranes (negative control), intense collagen staining was observed in the region below the epithelial layer and the collagen fibers were continuous. However, in the membranes treated with MVs, collagen was fragmented and thread like structures were observed indicative of collagen degradation. The collagen degrading effect could be attributed to the activity of proteases present in MVs since this could be attenuated when the chorio-decidual tissue was incubated with MVs in presence of protease inhibitors (Fig 3B).

We next tested the mechanical properties of the fetal membranes following treatment with MVs. By AFM analysis (Fig 3C), as compared to liposome challenge (2.85 ± 0.66 kPa); treatment of mouse chorio-decidual membranes with MVs revealed a significant reduction in the stiffness of the membranes (1.19 ± 0.03 kPa). This detrimental effect of MVs was not observed in presence of protease inhibitors as compared to MV treatment, incubation of MVs in presence of protease inhibitors had significantly higher stiffness (2.24 ± 0.04 kPa). Significant reduction in the stiffness was also observed when the membranes were treated with collagenase (1.40 ± 0.09 kPa) that was used as positive control (S2 Fig).

### GBS MVs anterogradely move in the mouse reproductive tract

**Fig 4:**
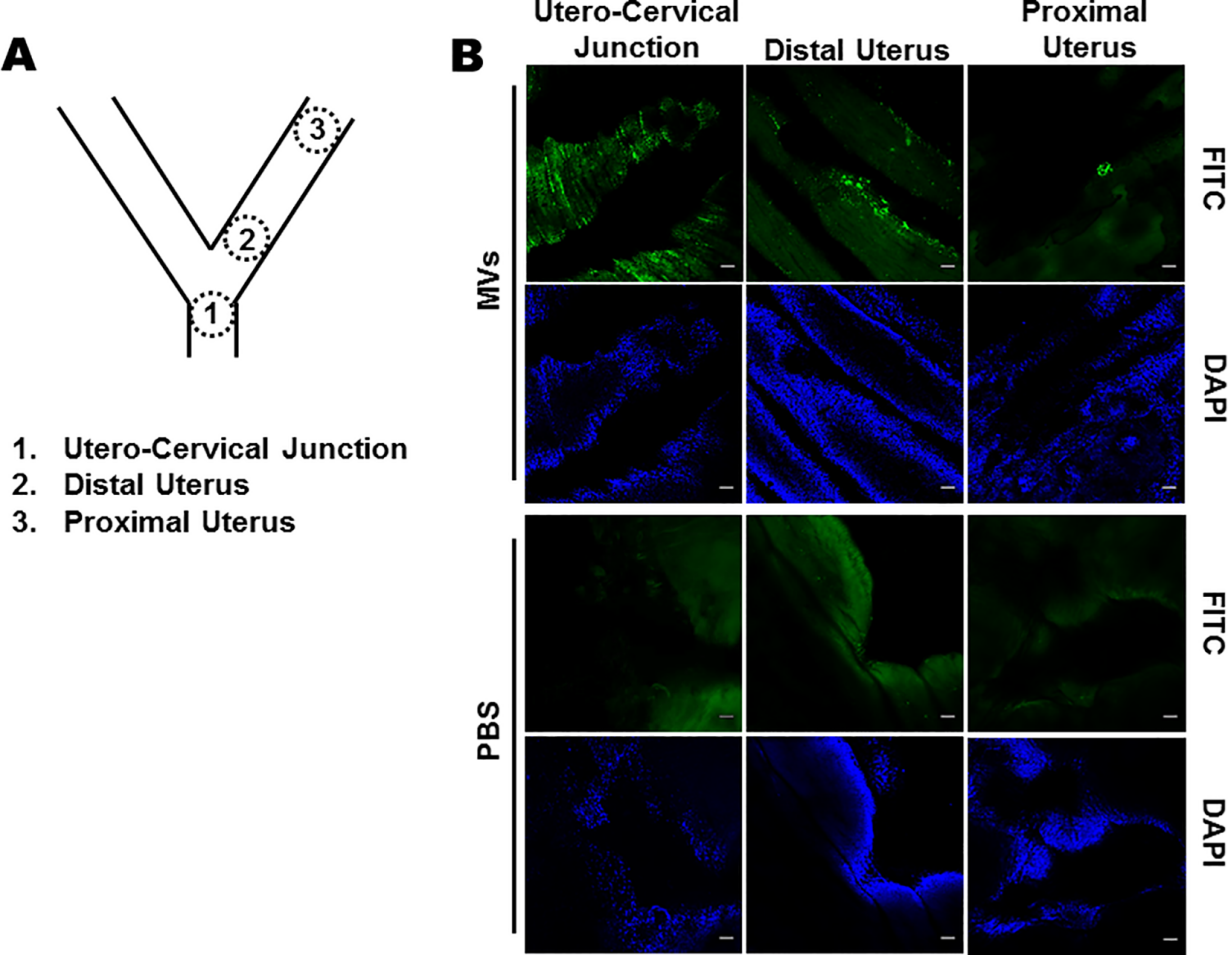
Anterograde transport of GBS MVs in female reproductive tract. **A.** Schematic diagram of female reproductive tract in mice. Areas denoted were visualized in the confocal microscope. **B.** FITC labelled GBS MVs (100 μg) were vaginally instilled and the reproductive tract (cervix to uterus) were collected after 6 h. Tissues were briefly fixed in paraformaldehyde and visualized under a confocal microscope. Bright green fluorescent spots above the background auto flourescence (as estimated in PBS injected animals) were considered to be derived from MVs. The experiment was done thrice. Representative images at the utero-cervical junction, distal uterus and proximal uterus are shown. Corresponding DAPI stained images are also shown. Scale bar; 50 μm.

To test if the MVs in the vagina can travel anterogradely in the uterus, FITC labeled MVs from GBS strain A909 were instilled in mouse vagina and 6 h later, the uteri were imaged using confocal microscope. Bright fluorescent signals were detected abundantly at the utero-cervical junction where the MVs seemed to be lodged in the folds and crevices of the tissue. Intense fluorescent signals were also detected in the distal uterus where MVs appeared to be layered on to the cells. In the anterior most segment of the uterus, although less frequent, high intensity signals were detected as clusters lodged on the walls of the uterus. These signals were specific to FITC labeled MVs as no such fluorescent signals were detected in the PBS injected controls (Fig 4). These observations imply that GBS MVs could anterogradely move along the female reproductive tract and reach distant sites.

### GBS MVs lead to disruption of amniotic epithelium and reduction in collagen *in vivo*

To determine the *in vivo* effect of MVs, we injected MVs from strain A909 in individual mouse fetal sacs and 24 h post injection, mice were sacrificed and chorio-decidual membranes were collected. Histopathogically, in the tissues obtained from MV injected amniotic sacs, extensive disruption of chorio-decidual epithelium and cell sloughing was observed; no such changes were seen in tissues derived from PBS or BSA entrapped liposomes injected controls.

**Fig 5:**
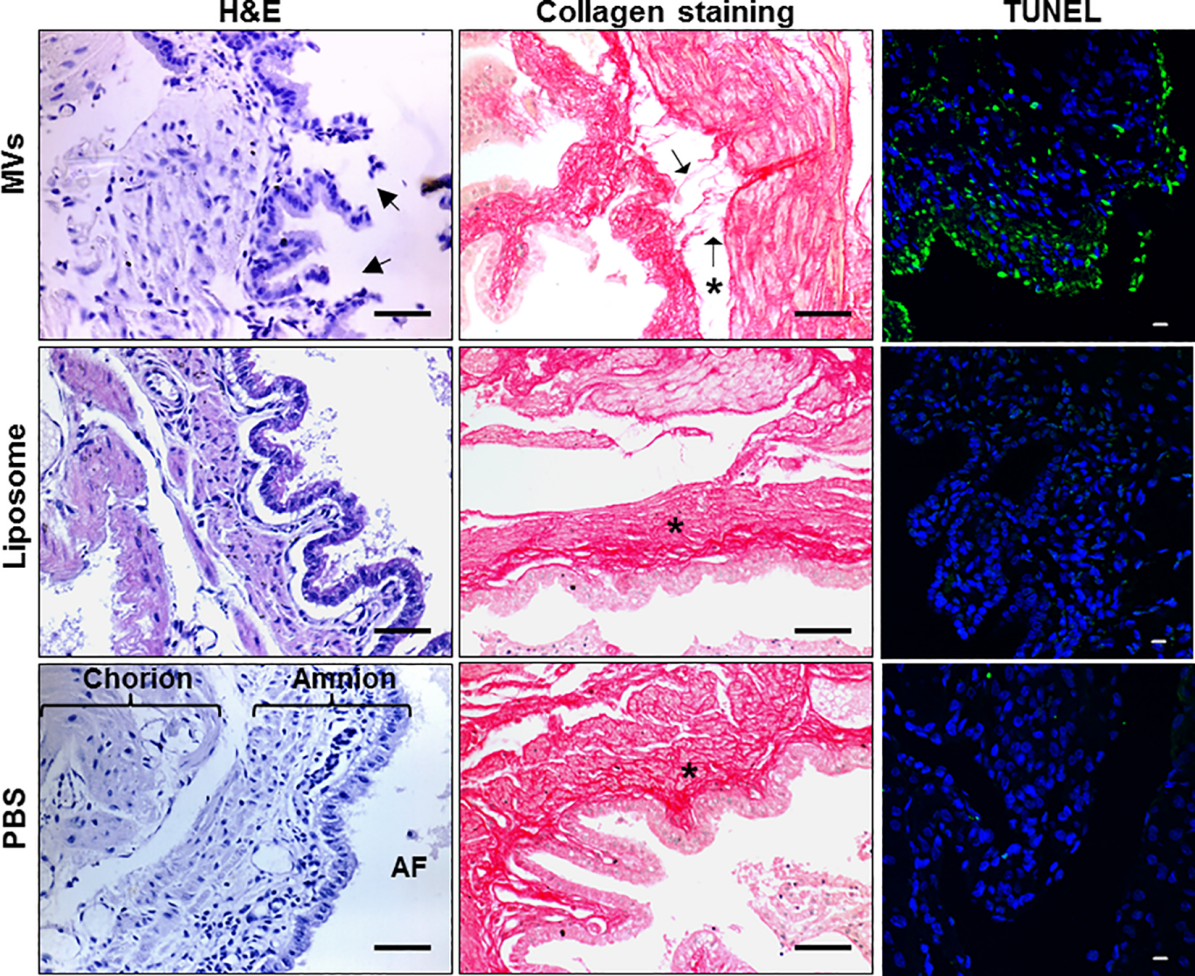
Intra-amniotic injections of GBS membrane vesicles cause tissue damage in the chorio-decidua. On E14.5 each fetus was injected with MVs or BSA encapsulated liposomes or PBS in the amniotic sac and after 24 h chorio-decidua were collected. Paraffin sections were stained with haematoxylin and eosin (H&E) and for collagen using Picrosirius red (scale bar; 200 μm) or processed for detection of apoptosis by TUNEL (scale bar; 10 μm). Arrows indicate disruptions in the epithelial cell lining (in H&E images) and collagen fiber disintegration (in collagen staining images) in MV injected tissues. Chorion, amnion and amniotic fluid (AF) are designated in the H&E image of PBS injected tissue. ‘*’ designates basement membrane in collagen staining images.

The collagen distribution in the mice feto-maternal tissues was studied histologically using Picrosirius red. In the controls (PBS or liposome injected groups), bundles of collagen fibers were detected all along the length of the sub-epithelial layer. In the tissues obtained from MV injected sacs extensive collagen fragmentation was observed, the collagen bundles appeared disrupted and a large number of disrupted collagen fibers were detected in the sub-epithelial zone (Fig 5).

We next examined the effect of GBS MVs on apoptosis in chorio-decidual membrane. Large numbers of TUNEL positive cells were detected along the entire area of the chorio-decidua derived from amniotic sacs injected with MVs indicative of extensive apoptosis. Very few apoptotic cells were observed in the tissues derived from liposome or PBS injected sacs (Fig 5). Similar apoptotic effects were observed in HeLa cells following treatment with GBS MVs *in vitro* (S3A Fig).

### Intra-amniotic injections of GBS MVs lead to leukocyte infiltration and chorio-amnionitis like features

**Fig 6:**
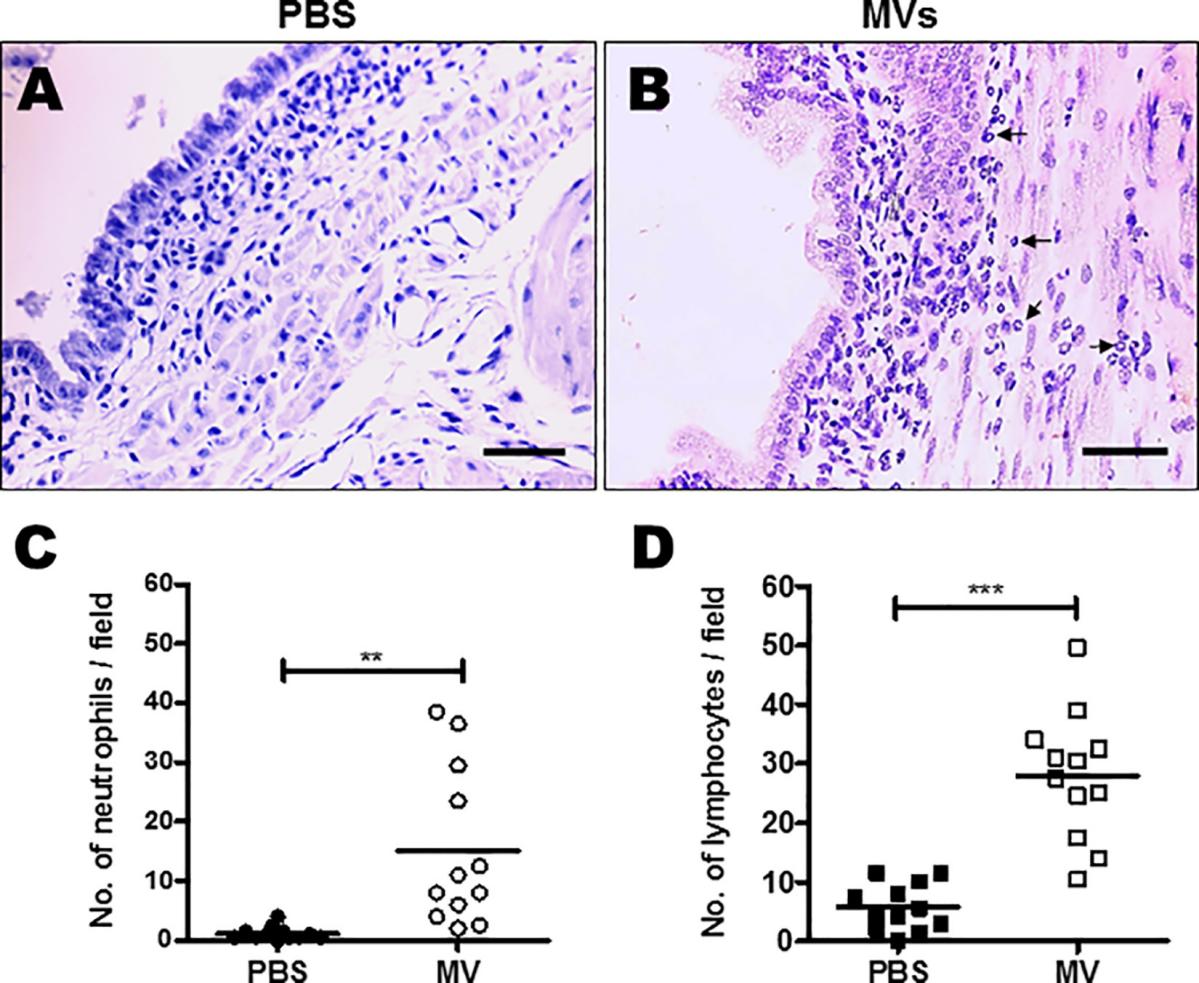
Intra-amniotic GBS membrane vesicles causes chorio-amnionitis. **A-B.** On E 14.5, each fetus was injected with PBS (**A)** or membrane vesicles (MVs) (**B**) in the amniotic sac. 24 h after injections, chorio-decidua were collected. Haematoxylin and eosin stained sections were examined for features of chorio-amnionitis. Arrows in B indicates leukocytes. Scale bar; 200 μm. **C-D.** Number of neutrophils (**C**) and lymphocytes (**D**) present in chorio-decidual tissue were enumerated across different fields as described in the methods. Statistical analysis was performed using Students t-test; ***p < 0.001; **p < 0.005.

**Fig 7:**
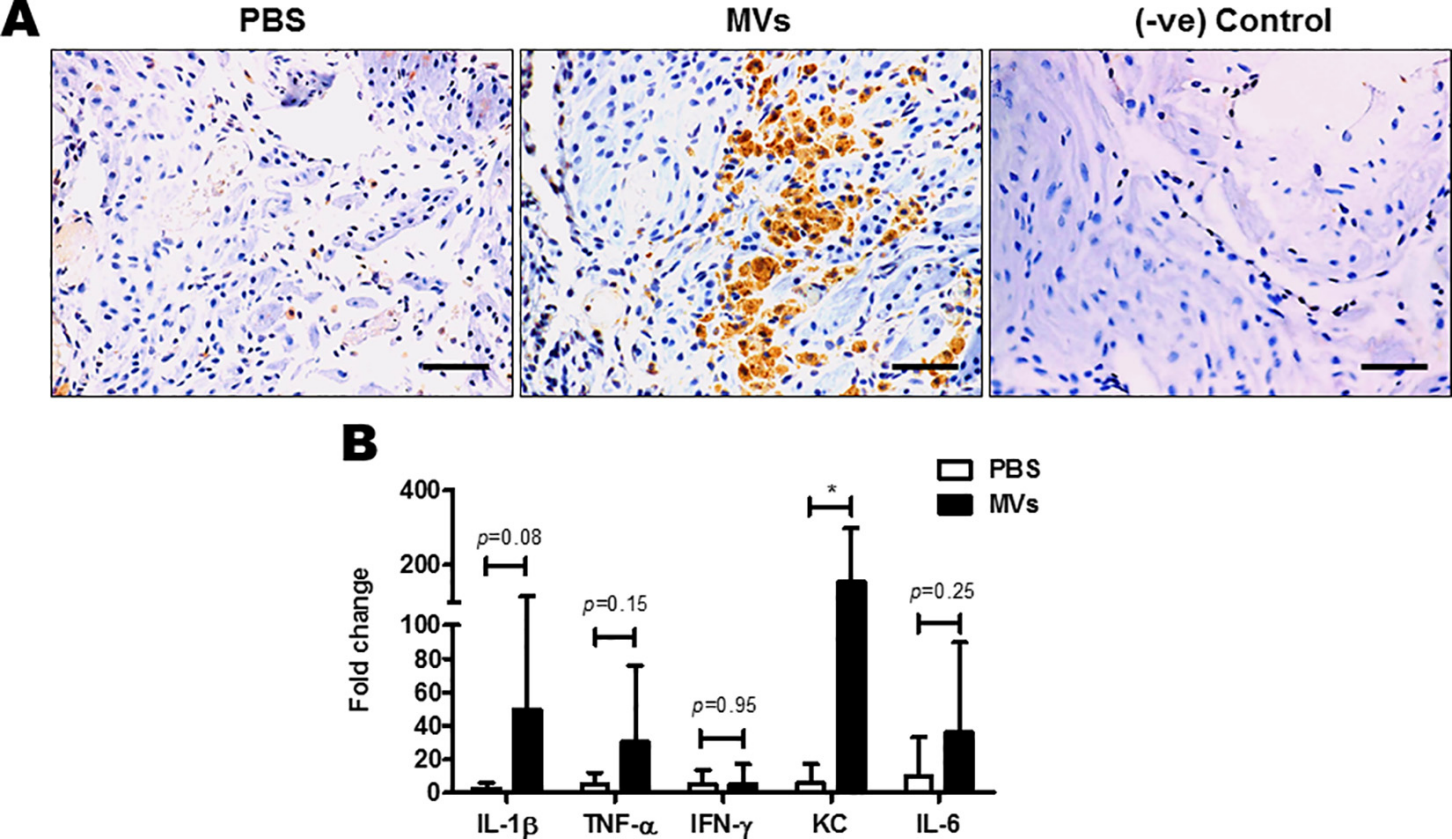
Intra-amniotic injections of GBS membrane vesicles cause inflammatory changes in the chorio-decidua. **A.** On E14.5, each fetus was injected with either PBS or membrane vesicles (MVs) in the amniotic sac. 24 h after injections, chorio-decidua were collected. Paraffin sections were stained using an antibody against the macrophage marker F4/80 Ab. Dark brown staining is for F4/80, indicating presence of macrophages.–ve Control implicates no non-specific binding of the secondary Ab. Scale bar; 200 μm. **B.** mRNA levels of IL-1β, TNF-α, IFN-γ, KC and IL-6 was determined by qRT–PCR on total RNA isolated from decidua, 24 h post injection with MVs (n = 8). Transcript levels were normalized to 18s rRNA and expressed as fold change compared with tissues injected with PBS only. Statistical analysis was performed using two-way ANOVA (Bonferroni test); *P < 0.05.

Examination of the chorio-decidual tissue derived from amniotic sacs injected with MVs of GBS strain A909 revealed infiltration of neutrophils and lymphocytes underneath amniotic epithelium, a hallmark of chorio-amnionitis (Fig 6A and 6B). Scoring of inflammation revealed presence of significantly higher numbers of neutrophils and lymphocytes in tissues derived following MV injections compared to PBS or liposome injected tissues (Fig 6C and 6D and S4A and S4B Fig). A large number of F4/80 positive macrophages were also seen infiltrated in case of MV injected tissues (Fig 7A). In case of control sacs (injected with PBS or liposomes) relatively fewer F4/80 stained macrophages and neutrophilic/leukocytic infiltration was observed (Fig 7A and S4 Fig).

### GBS MVs promote extensive inflammation in the chorio-decidua

We next examined if GBS MVs induce an inflammatory response in the host tissues. For this we injected MVs from GBS strain A909 in individual mouse fetal sacs on E14.5 and 24 h later the mRNA levels for selected inflammatory cytokines were estimated by qPCR in the decidua. In the decidua derived from MV injected sacs there was a significant increase in the transcript levels of *Kc*, *Il-1β*, *Il-6* and *Tnf-α* compared to decidua derived from PBS injected amniotic sacs (Fig 7B). However, the levels of *Ifn-γ* remained unchanged between the two groups. Amongst all the studied cytokines, a maximum increase was observed in the mRNA levels of *Il-1β* and *Kc*. The inflammatory response was specifically driven by injected MVs, as no difference in transcript abundance of inflammatory cytokines was observed in tissues derived from liposome compared to PBS injected sacs (S4G Fig).

### GBS MVs lead to preterm birth and fetal demise

**Fig 8:**
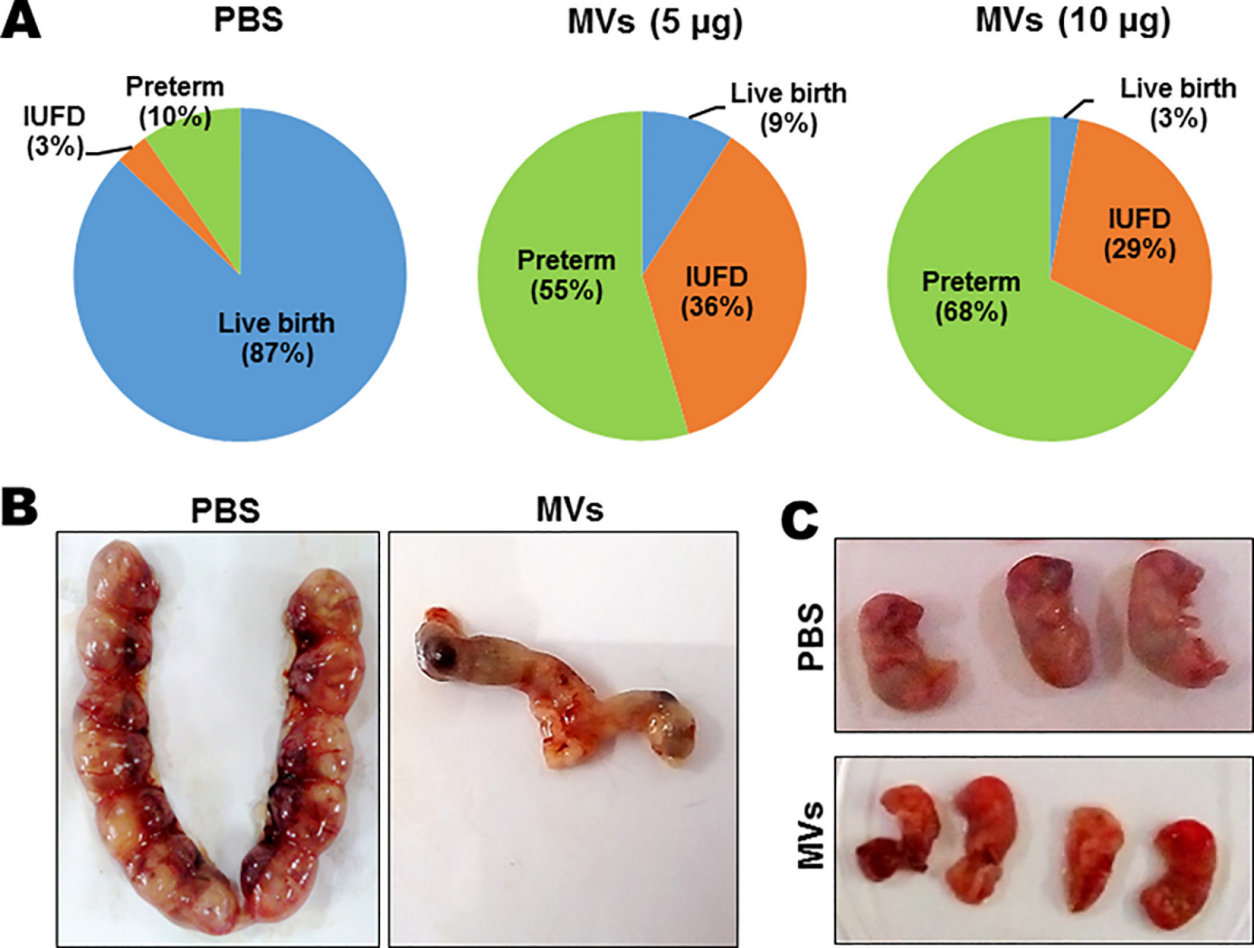
Intra-amniotic injections of GBS membrane vesicles lead to preterm birth and fetal demise. **A.** Fetal outcomes of intra amniotic injections with GBS membrane vesicles (MVs). Mice on E14.5 were injected with PBS or MVs (at 5 or 10 μg of MV protein/sac) and observed for 72 h. The number of live births or pups delivered preterm (before E20) or intrauterine fetal death (IUFD) were recorded and represented as pie charts. **B.** Representative images of uteri of mice injected with MVs on E18.5 (72 h after injection). **C.** Representative images of pups on E18.5 derived from uteri or delivered after intrauterine injection of PBS or MVs on E14.5.

Considering our findings on the widespread effect of GBS MVs on fetal membrane components we tested if MVs from GBS strain A909 are capable of inducing preterm birth. For this purpose amniotic sacs of mice were injected with either 5 or 10 μg of GBS MVs or PBS and animals were monitored every 6 h up to 72 h for signs of preterm birth (vaginal bleeding, pups in cage). Administration of 5 μg and 10 μg of MVs in the amniotic fluid resulted in preterm births (by day 18 of pregnancy) of 55% and 68% of fetuses, respectively. Only 10% of such events were observed in the PBS treated group. Along with preterm birth, increased frequency of intrauterine fetal death (IUFD, defined as resorption or presence of a macerated fetus in amniotic sac) was observed in the group treated with GBS MVs as compared to PBS treated controls (36% and 29% vs 3%) (Fig 8A and S2 Table). Interestingly, in the groups where MVs were injected, fetuses were either resorbed or delivered by 48 h. Moreover, the delivered fetuses from MV injected sacs exhibited significant reduction in size and abnormal morphology (Fig 8C). Collectively these suggest that GBS MVs lead to fetal demise, preterm birth and cause fetal injury.

## Discussion

Membrane vesicles are bilayered structures, found to be secreted almost ubiquitously by gram negative bacteria and a large number of gram positive bacteria [24,25,28,29,31–34]. In the current study, we demonstrate for the first time that GBS (a gram positive bacteria) is capable of secreting MVs in a strain independent manner. Employing different microscopic techniques we could not only detect the presence of vesicles in GBS culture supernatant, but also demonstrate the budding of vesicular structures from GBS surface. Interestingly, similar budding structures from GBS surface resembling MVs have been reported in GBS colonized in the murine genital tract, implying that GBS could also produce MVs *in vivo* [35]. We isolated and characterized these MVs both physically and biochemically and examined their pattern of interaction with host cells in order to explore their potential contribution in GBS pathogenesis. Intriguingly, we could extract DNA and amplify the *cfb* gene, an important virulence factor of GBS, using the MV DNA as template. Occasionally, DNA has been found to be associated with MVs from other microorganisms such as *P*. *aeruginosa* [36], *S*. *vesicuolsa* [37] and *C*. *perfringens* [26]. Though DNA is detected in GBS MVs, we believe this DNA is fragmented as we could only detect the *cfb* gene, but genes representing other virulence factors (*cylE*, *pepB*, *zooA*) or housekeeping gene (*gapN*) could not be amplified. This implies that the DNA packaged in MVs is perhaps not a random phenomenon but may involve a regulated mechanism. While the functional significance of the DNA packaging in MVs is yet unknown, since the MVs fuse with the host or other bacterial cells, these might act as carriers of DNA from one cell to another [38]. It will be of interest to determine if the MV DNA specifically enters the cells to induce a pathological activation of host nuclease machinery.

Proteomic analysis of the GBS MV proteins revealed presence of numerous ECM degrading enzymes. The preferential packaging of ECM degrading enzymes compared to other abundant membrane proteins implies presence of a selective sorting mechanism. Such a sorting mechanism have been identified in *P*. *gingivalis* which not only facilitates preferential packaging of important virulence factors but also enables it to exclude other abundant outer membrane proteins from the cargo [39]. Similar enrichment of acidic glycosidases and proteases were observed in *Bacteroides* species suggesting presence of a species specific machinery devoted to selectively pack proteins into the vesicles to do specific jobs, in this case securing nutrients for the benefit of the whole bacterial community present in the microbiota [40].

The presence of various ECM degrading enzymes in GBS MVs therefore points towards its significant role in GBS pathogenesis. Indeed, we observed that the MVs could not only interact with HeLa cells extracellularly but also internalize and cause cell death. These observations prompted us to hypothesize that the presence of various virulence factors and ECM degrading proteins in GBS MVs might lead to tissue degradation and cell death that may contribute to PPROM and preterm births. We next performed a series of experiments to investigate this hypothesis.

The fetal membranes (amnion and chorion) rest upon a collagenous basement membrane of type II and IV collagen and beneath this layer lays a fibrous layer that contains collagen types I, III, V, and VI. Collagen, therefore provides major structural strength for the membranes and degradation of it leads to loss of membrane integrity [41–43]. The enrichment of ECM degrading proteases, coupled with the presence of gelatinolytic activity in GBS MVs suggested that these might cause loss of fetal membrane integrity by ECM degradation. Indeed, *ex vivo* treatment of chorio-decidual membrane with GBS MVs led to collagen fragmentation. This collagen fragmentation had profound effect on fetal membrane integrity as the stiffness of the membranes challenged with MVs were significantly reduced. It is likely that during pregnancy, GBS via its MVs lead to collagen degradation and reduction in tissue stiffness which together would resist further expansion of the amniotic sac, a prerequisite to accommodate the growing fetus. Thus our findings suggest that GBS MVs lead to loss of ECM and weaken the amniotic membrane making it susceptible to rupture upon pressure from the growing fetus.

Epidemiological data suggest that colonization of vagina and cervix with GBS increases the probability of chorio-amnionitis. We suspected that the MVs produced by GBS while colonizing can move upwards in the female reproductive tract and affect cells and tissue at distant sites. Budding structures resembling MVs have been observed in GBS colonized in the murine genital tract [35]. Extending this data, herein we show that GBS MVs when vaginally instilled could traverse anterogradely upto anterior most segment of the uterus. Recently, it has been shown that GBS mutant for hyaluronidase is not capable of ascending infection in a mouse model [44]. Since, hyaluronidase is also enriched in our GBS MV preparations; it is possible that it could aid ascend of MVs into the female tract. While it needs to be demonstrated that like GBS, MVs can also anterogradely transport into the gravid uterus; it is tempting to hypothesize that GBS colonization at a distant site such as vagina can affect the feto-maternal tissues in the uterus by the virtue of secreting MVs.

We next asked if GBS MVs have any pathogenic effects *in vivo*. Corroborating our *ex-vivo* observations; we observed that instillation of GBS MVs intra-amniotically led to extensive disruption of the chorio-decidual structure. This disruption is also associated with collagen fragmentation which could be due to the proteases present in the MVs. Beyond collagen, extensive alterations in the expression of genes encoding for extracellular matrix proteins has been reported in chorio-decidua of rhesus monkeys infected with GBS [16]. While it will be of interest to see the alterations in other ECM molecules in response to MVs, we believe that the changes in extracellular matrix and collagen degradation would contribute to the mechanical weakening of the tissue resulting in the loss of membrane stiffness. Indeed we did see a reduction on stiffness of the chorio-decidua tissue challenged with MVs *ex vivo*. It will be of interest to test the effects of MVs derived from GBS mutants for various ECM degrading enzymes and determine the mechanistic basis of this phenomenon. Along with loss of collagen, we also detected extensive apoptosis in the chorio-decidua derived from sacs injected with MVs. These observations suggested that coupled with mechanical weakening, cell death might further contribute to loosening of the membrane. Such tissue damage by MVs could make the membrane prone to rupture leading to PPROM.

A leading feature of PPROM and preterm births due to inflammation is chorio-amnionitis. Chorio-amnionitis is acute inflammation of the fetal membrane and chorion which is typically caused due to ascending microbial infection especially in case of infection with genital mycoplasmas [45]. Histologically, chorio-amnionitis is characterized by leukocyte infiltration in the chorion/amnion and the decidua. In the context of GBS, *in vivo* administration of the live bacteria in pregnant mice led to histopathologic characteristics resembling chorio-amnionitis [16]. Herein for the first time we demonstrated that chorio-amnionitis can be caused by MVs even in absence of live bacteria. We observed that intra-amniotic administration of GBS MVs caused extensive leukocytic infiltration in the subchorion layer and in decidua resembling chorio-amnionitis. Beyond leukocytic infiltration, the decidua of mice injected with GBS MVs also had macrophage infiltration. Moreover, these vesicles also induced inflammatory cytokines such as, *Kc*, *Il-1β*, *Il-6*, and *Tnf-α* in the decidua. Levels of these pro-inflammatory cytokines are also known to be elevated in the amniotic fluid of patients in independent contexts of GBS infections and PPROM [16,46]. Since HeLa cells also showed similar response upon treatment with MVs (S3B Fig), we believe that the elevation in levels of these cytokines in the decidua may not be exclusively due to higher numbers of immune cells present in these tissues, but because of the ability of MVs to generate an inflammatory response in the decidual cells. We therefore conclude that GBS by the virtue of production of MVs activates the host immune system which might trigger the immune cell homing and activation leading to chorio-amnionitis. These observations are novel as for the first time we have shown that even in absence of active infection in the chorio-decidua, features resembling clinical chorio-amnionitis could be mimicked by MVs. Clinically this observation is highly relevant as 50–80% women with chorio-amnionitis do not have bacteria in their amniotic fluid or the decidual tissue [47–50]. Based on our findings we can hypothesize that MVs secreted by the pathogens residing in lower genital tract may be responsible for cases with unexplained chorio-amnionitis. It would be imperative to study the presence of MVs in amniotic fluid and chorio-decidual tissues of woman with culture negative chorio-amnionitis.

Since, GBS MVs lead to tissue damage, inflammation and chorio-amnionitis, resulting in mechanical weakening of the fetal membrane, we finally asked if GBS MVs can lead to premature birth. Indeed, we observed that intra-amniotic injection of GBS MVs lead to preterm delivery. More than 50% of mouse pups were delivered preterm (almost 2 days prior to their expected day of delivery) when challenged with MVs. Finally, we also observed that like live GBS [17], the MVs are also highly pathogenic to the fetus. Almost 30–40% of fetuses died *in utero* amounting to resorption and some fetuses were still born. The fetuses that were stillborn or recovered from uterine sacs after GBS MV challenge, were smaller and had major damage to its organs. These results together imply that GBS MVs can cause IUFD or preterm births. To our knowledge this is the first report describing the direct role of membrane vesicles produced by any pathogenic bacteria in disease pathogenesis. In the light of the fact that intrauterine infections can lead to autism like changes in the brain [51], it is possible that GBS MVs might have a similar effect. It will be of interest to study the effects of sub-lethal dose of GBS MVs on fetal development and physiology.

In summary, the results of the present study have shown that GBS MVs cause host cell death, membrane weakening and inflammation of the feto-maternal interface which causes preterm birth and IUFD. Coupling this with the fact that pathogen derived vesicles can function as vehicles for long distance delivery of virulence factors [52], our results imply that the production of extracellular membrane vesicles serves not only as a tool for secretion but also arms GBS with an additional weapon by which while colonizing it can orchestrate events at distant sites including the fetal membrane. Our findings provide a plausible explanation for the occurrence of PPROM and premature delivery in woman with chorio-amnionitis without detectable bacteria in their amniotic fluid or the decidual tissue. We conclude that GBS utilizes MVs as a surrogate to spread its virulence factors in the host which are responsible for the clinical features of GBS infection during pregnancy. Prevention of vesicle biogenesis may therefore be a viable therapeutic option to prevent GBS mediated preterm birth.

## Materials and Methods

### Ethics statement

All the experimental work on animals was done as per the guidelines of the Committee for the Purpose of Control and Supervision of Experiments on Animals (CPCSEA), India. The study protocol has been reviewed and approved by the Institutional Animal Ethics Committee of National Institute for Research in Reproductive Health (NIRRH) under the project number 10/15.

### Bacterial strains and isolation of MVs

*S*. *agalactiae* strains A909 (serotype IA), COH1 (serotype III), NEM316 (serotype III) and 2603V/R (serotype V) (kindly provided by Dr. Lakshmi Rajagopal, Department of Pediatric Infectious Diseases, University of Washington School of Medicine, Univ. of Washington, Seattle, USA) were cultured in Todd Hewitt broth (THB) at 37^○^C. Unless otherwise stated MVs from GBS strain A909 was used for further experiments. MVs were purified from growing GBS cultures as per the standard protocol used for many other bacteria [25,27,32,53,54]. Briefly, GBS cells grown upto optical density (OD_600nm_) 1.2, were harvested by centrifugation (12,000 x g, 30 min, 4^○^C) and the supernatant was passed through 0.22μm filter. The filtrate was concentrated using Amicon ultrafiltration system (10 kDa) and ultracentrifuged (150000 x g, 3 h, 4^○^C) to pellet down MVs. The MV pellet was resuspended in PBS (pH 7.2) and protein content was determined by Bradford assay following lysis of MVs by 0.05% Triton X-100.

To quantitate the number of MVs produced from GBS culture, MVs were labeled with 20 μM of Vybrant DiO cell labeling solution (Molecular Probes) and quantified by flow cytometry (FACS Aria II, BD Biosciences, USA) using fluorescent counting beads (CountBright Absolute counting beads; Invitrogen) as standards as described earlier [25]. The varying diameters and size distribution of MV preparations were measured using a Goniometer (Brookhaven Instrument Co., USA) as described earlier [55].

### Electron microscopy and atomic force microscopy for GBS MVs

For Scanning Electron Microscopy (SEM), GBS cells were air dried, desiccated overnight and visualized with Field Emission Gun Scanning Electron Microscope (JEOL, USA) at an accelerating voltage of 5 kV. For Transmission Electron Microscopy (TEM), MV samples were applied to Formvar/Carbon film coated 200-mesh copper grids (Pacific Grid-Tech) and negatively stained with 2.5% uranyl acetate followed by visualization under Transmission Electron Microscope (FEI Technai, USA) (120 kV). For Atomic Force Microscope (AFM), bacterial suspension was loaded onto poly-L-lysine coated coverslips and visualized under an atomic force microscope (Asylum Research, USA) under contact mode at a scanning rate of 1 Hz using silicon nitride cantilevers.

### Fatty acid analysis

Lipids were extracted from intact GBS cells and MVs using protocol described earlier [25]. Following extraction, lipids were silylated using 1:1 ratio of N,O-Bis(trimethylsilyl)trifluoroacetamide (BSTFA) and pyridine at 75^○^C for 30 min. Silylated lipids were then analyzed using a GC-MS (Agilent, USA) fitted with a HP-5MS fused silica capillary column (30 m × 0.25 mm i.d., 0.25 μm film thickness) with Helium as carrier gas (flow rate 1ml/min). Identification of compounds was based on mass spectra including comparison to a library (NIST).

### Protein analysis

150 μg of MV proteins were separated on 12% SDS-PAGE. After staining with Coomassie Brilliant Blue R-250 (CBB), bands were excised and processed for in-gel trypsin digestion [56]. The eluted oligopeptides were co-crystallized with CHCA (5 mg/ml) and spectra were acquired using MALDI-ToF mass spectrometer (UltraFlex III, Bruker Daltonics). Mascot (Version 2.2.04, Matrix Science) searches were conducted using the NCBI non-redundant database with the following settings: 1 missed cleavage; Carbamidomethyl on cysteine as fixed modifications, methionine oxidation as variable modification and 100 ppm error (150 ppm error for band No. 4). A match with *S*. *agalactiae* protein with the best score in each Mascot search was accepted as successful identification (*p*< 0.05). Subcellular locations were predicted by LocateP database and confirmed by pSORT and TMHMM algorithms.

### Isolation and analysis of nucleic acid from MVs

MVs were initially treated with 50 μg/ml DNaseI in presence of 10 mM MgCl_2_ at 37°C for 1 h to remove any surface bound DNA. Following heat inactivation (10 min at 80°C) and lysis of MVs using Triton X-100 (0.05%) at 37⁰C for 30 min, DNA was extracted by phenol-chloroform-isoamyl alcohol, precipitated using ammonium acetate as described earlier [26]. The purified DNA was quantified and used for PCR using primers specific for *cylE*, *cfb*, *pepB*, *zooA* and *gapN* genes (S1 Table). Genomic DNA isolated from GBS strain A909 served as control template.

### Cell culture, internalization of MVs and cytotoxicity

HeLa cells (procured from National Center for Cell Science, Pune, India) were grown in DMEM media (Invitrogen) supplemented with 10% fetal bovine serum (Invitrogen) at 37^○^C in 5% CO_2_. HeLa cells were incubated with increasing protein concentrations of MVs (10 μg/ml to 300 μg/ml) for 24 h and the viability was assessed using MTT assay kit (HiMedia, India).

To study internalization, MVs were labeled with FITC (Sigma) in 0.1 M sodium carbonate buffer (pH 9.0) for 1 h at room temperature and washed to remove unbound FITC [57]. FITC labeled MVs (30 μg MV protein) were then allowed to interact with HeLa cells for 6 h. After washing to remove unbound MVs, cells were fixed and stained with mouse polyclonal anti-FITC antibody (Invitrogen) followed by an anti-mouse secondary antibody conjugated to AlexaFluor 555 (Invitrogen). Images were acquired with an oil immersion Plan-Apochromat 63X/1.4 NA objective using a confocal laser scanning microscope (Zeiss).

### Gelatin zymography

10 μg of MV proteins were separated (150 V, 5 h, 4°C) on 12% SDS-PAGE with 0.1% co-polymerized gelatin. After treatment in 1% Triton X-100, gelatinolysis was promoted by further incubation for 16 h at 37°C in developing buffer (50 mM Tris-HCl, 50 mM Tris base, 200 mM NaCl, 5 mM CaCl_2_, 0.02% Brij 35, pH 7.6). The gel was then stained with Coomassie Brilliant Blue R-250 and destained to intensify the digestion halos.

### Preparation of liposome

BSA entrapped liposomes were prepared by thin film hydration method [58]. Briefly, phosphatidylcholine (10 mg) was dissolved in a mixture of chloroform and methanol (2:1) and the solvent was evaporated under vacuum using a rotary evaporator to form a thin layer of lipid film in a round bottom flask. The dried lipid film was hydrated using 1 ml of PBS containing BSA (10 mg/ml) at 45°C for 1 h to form multilamellar vesicles (MLV). The liposome suspension was then sonicated using a probe sonicator at 40% amplitude to obtain unilamellar vesicles.

### Atomic force microscopy for tissues

Chorio-decidual membrane was collected from pregnant mice on E14.5. Immediately after dissection, the membranes were incubated in DMEM (Invitrogen) containing 10% FBS (Invitrogen) at 37°C and treated for 24 h with either PBS, or BSA entrapped liposomes (100 μg/ml) or MVs (100 μg/ml) in presence or absence of protease inhibitor cocktail (Sigma). Collagenase (10 μg/ml) was used as positive control. Following incubation, the membranes were washed, carefully spread on a slide layered with double adhesive tape, allowed to air dry for 5 min. Subsequently, the membranes were hydrated with PBS, and their mechanical properties were probed with an Atomic Force Microscope (AFM). A 5 μm diameter spherical probe with a nominal spring constant of 32.66 pN/nm was used. Using a custom-written code, force-indentation curves were fitted with Hertz model to estimate the Young’s modulus of elasticity of each of the membranes. Each sample was probed randomly multiple number of times (greater than 50) at multiple different positions to estimate average stiffness of the membranes.

### Anterograde transport of MVs

The vagina of mice in estrus phase were flushed thrice with 40 μl of 0.2% Triton X-100 in 0.9% saline followed by 40 μl 0.9% saline. MVs were labeled with FITC as described earlier [57] and 100 μg of FITC labeled GBS MVs in 100 μl PBS were vaginally instilled in 25–30 μl aliquots using a micropipette. Control mice received PBS only. After 6 h mice were euthanized and the reproductive tract (cervix to uterus) was collected. Tissues were briefly fixed in 4% paraformaldehyde and mounted on slides with Vectashield containing DAPI (Vector Laboratories). Images of different parts of the tissue such as utero-cervical junction, distal uterus and proximal uterus were captured with an oil immersion Plan-Apochromat 40X/1.3 NA objective of a confocal laser scanning microscope.

### Intra-amniotic administration of MVs

For pregnancy-outcome experiments, C57BL6/J- were bred and maintained at the Experimental Animal Facility of NIRRH under constant temperature and 12 h light and dark cycles were used. Female mice in estrus were impregnated naturally by a male of proven fertility and mating was confirmed by the presence of a vaginal plug (E0.5). Intra-amniotic injections were performed on E14.5 of a 19–20 d gestation. Briefly, animals were anesthetized and a 1.5-cm midline incision was made in the lower abdomen. The mouse uterus is a bicornuate where the fetuses are arranged in a “beads-on-a-string” pattern. Both the horns were exposed and individual fetal sacs were injected with 100 μl of MVs (5 or 10 μg protein) or equivalent amount of PBS or BSA-liposomes. After injection the uterus was returned to the abdomen, muscle and skin layers were sutured and dams were returned to their cages and monitored on regular intervals. Surgical procedures lasted ∼10 min and post-operative care was taken as per standard protocols at NIRRH. Delivery of one or more pups in the cage or lower vagina within 48 h was considered preterm.

For collection of tissues, animals were euthanized 24 h after surgery. The inoculated horns were incised longitudinally along the anti-mesenteric border. Gestational tissues (full-thickness biopsies from the middle region) and fetal membranes were harvested and frozen in Trizol reagent (Invitrogen) and stored at −80°C for RNA extraction or fixed in 4% paraformaldehyde for histopathology. Fixed tissues were paraffin embedded and 5 μm thick paraffin sections were collected on poly-lysine coated glass slides and processed for routine hematoxylin and eosin staining.

### RNA extraction, cDNA synthesis and qRT-PCR

RNA from HeLa cells or mouse tissue was isolated using Trizol reagent as detailed previously [59]. RNA was treated for 30 min with DNaseI to remove any DNA contamination and processed for reverse transcription. The details of qRT-PCR have been described previously [59]. Briefly, 1 μg of RNA was reverse-transcribed using SuperScriptTM First-Strand Synthesis System (Invitrogen). The cDNA was further used for quantitative RT-PCR (qRT-PCR) for various cytokines. β-actin (for HeLa cells) and 18S (for mouse tissues) were used as the house keeping genes. The sequences of the primers are as mentioned in S1 Table. Care was taken to design primers that spanned an intron to eliminate any amplification due to genomic DNA contamination. The specificity of amplicons was confirmed by performing dissociation / melt curve analysis. Only those primer pairs that resulted in a single sharp melt peak with a consistent melt temperature were included in the study. The relative changes in the expression of above genes was analyzed by 2^-ΔΔCt^ method [60].

### Quantification of leukocytes

Hematoxylin and Eosin stained slides of the chorio-decidua were examined under 40X objective for neutrophils and lymphocytes. The cells were identified based on their morphology. The number of neutrophils and lymphocytes per field were counted in 8–10 random fields per section. Five random sections from each fetus were analyzed. The analysis was done in three biological replicates.

### Immunohistochemistry

Immunohistochemistry on tissue sections was performed as detailed previously [61]. For detection of macrophages, paraffin embedded 5 μm sections of the chorio-decidual membrane (with or without MV treatment) were stained with rabbit polyclonal anti-F4/80 Ab (1:100; Santa Cruz Biotechnology) and detected using HRP conjugated goat anti-rabbit secondary antibody (1:100; Dako) and 3.3’ Diaminobenzidine with H_2_O_2_. Sections were counterstained with hematoxylin and mounted in DPX.

### Collagen staining

Total collagen staining in chorio-decidual membrane sections were performed using Picrosirius red [62]. Paraffin sections were deparaffinized, rehydrated in graded methanol series and stained with hematoxylin followed by 0.5% Direct Red 80 (Sigma) prepared in picric acid. Following dehydration with 100% methanol and clearing in xylene, the slides were mounted with DPX.

### TUNEL

Induction of apoptosis by GBS MVs was analyzed by *In Situ* Cell Death Determination Kit (Roche) according to manufacturer’s protocol. Briefly, following deparaffinization, tissue sections were rehydrated and digested using proteinase K. After permeabilization with 0.25% Triton X 100, the sections were incubated with terminal deoxynucleotidyl transferase and fluorescein-dUTPs for 1 h at 37°C. Following washing to remove unbound dUTPs, the sections were mounted with Vectashield containing DAPI (Vector Laboratories). Images were acquired with an oil immersion Plan-Apochromat 40X/1.3 NA objective of confocal laser scanning microscope (Zeiss).

### Statistical analysis

Graphpad Prism version 4.03 was used for statistical analysis. Statistical tests undertaken for individual experiments are mentioned in the respective figure legends. Statistical significance was accepted at *p*< 0.05.

## Supporting Information

S1 Fig GBS produces membrane vesicles in a serotype independent manner. **A.** Atomic force micrograph of GBS strain A909 cells demonstrating presence of spherical MVs (black arrowheads) of different sizes around the cells. **B.** SEM analysis of different GBS serotype strains, COH1, serotype III; NEM316, serotype III; 2603V/R, serotype V; demonstrating secretion of MVs similar to serotype IA strain A909. Scale bar; 1 μm (for COH1) and 100 nm (for NEM316 and 2603V/R). **C.** Growth curve of GBS strain A909. Growth of bacteria was analyzed by measuring optical density at 600 nm (OD_600_) at different time intervals. OD value corresponding to 1.2 (late exponential phase of growth) has been marked with a red circle.
(TIF)

S2 Fig Atomic Force Microscopy of mouse chorio-decidual membrane. Mouse chorio-decidual membranes (on E14.5) were treated with either PBS, MVs or collagenase (10 μg/ml) for 16 h and probed at multiple positions over a randomly selected region of 50 μm X 50 μm to estimate average stiffness. Force-indentation curves were fitted with Hertz model to estimate the Young’s modulus of elasticity. Experiments were performed thrice and bars represent standard deviation of the mean of one representative experiment. Statistical analysis was performed using one-way ANOVA (Tukey’s multiple comparison test); ***p < 0.001; **p < 0.005.
(TIF)

S3 Fig GBS MVs induce apoptosis and inflammation in HeLa cells. **A.** GBS MVs induced apoptosis in HeLa cells. HeLa cells were incubated with 100 μg/ml MVs for 24 h and stained with PI and/or Annexin V. Only Annexin V stained cells or PI and Annexin V double positive cells were analyzed by FACS and designated as early and late apoptotic cells, respectively. Experiments were performed thrice and bars represent standard deviation of the mean of one representative experiment. Statistical analysis was performed using one-way ANOVA (Tukey’s multiple comparison test); **p < 0.005. **B.** GBS MVs induced inflammatory response in HeLa cells. HeLa cells were incubated with 100 μg/ml MVs for 12 h and transcript levels of IL-1β, IL-6, IL-8 and β-actin were examined by qRT-PCR. Transcript levels were normalized to β-actin and expressed as fold change compared with cell treated with PBS only. Experiments were performed thrice and bars represent standard deviation of the mean of one representative experiment. Statistical analysis was performed using two-way ANOVA (Bonferroni test); ***p < 0.001.
(TIF)

S4 Fig *In vivo* treatment with BSA encapsulated liposomes does not induce chorio-amnionitis and inflammatory response. **A-D.** Intra-amniotic injections of PBS (**A, C**) or BSA encapsulated liposomes (**B, D**) were given to E14.5 day old pregnant mice and the embryos recovered on E15.5. Isolated chorio-decidua were fixed and stained with H&E as well as with anti-F4/80 Ab to detect presence of macrophages. Scale bar; 200 μm. **E-F.** Presence of neutrophils (**E**) and lymphocytes (**F**) were scored in different fields in chorio-decidual tissue that were injected with either PBS or liposomes. Statistical analysis was performed using Students t-test; ns, non-significant. **G**. mRNA levels of IL-1β, TNF-α, IFN-γ, KC and IL-6 was determined by qRT–PCR on total RNA isolated from mouse decidua, 24 h post injection with liposomes (n = 8). Transcript levels were normalized to 18s rRNA and expressed as fold change compared with tissues injected with PBS only. Statistical analysis was performed using two-way ANOVA (Bonferroni test); ns, non-significant.
(TIF)

S1 Table Oligonucleotides sequences. (DOCX)

S2 Table Fetal outcomes of intra amniotic injections with GBS MVs. (DOCX)
