# Supplementary figures and images for: Membrane Vesicles of Group B Streptococcus Disrupt Feto-Maternal Barrier Leading to Preterm Birth
Source: PLoS Pathog. 2016 Sep 1;12(9):e1005816. doi: 10.1371/journal.ppat.1005816 (PMC5008812; doi:10.1371/journal.ppat.1005816)

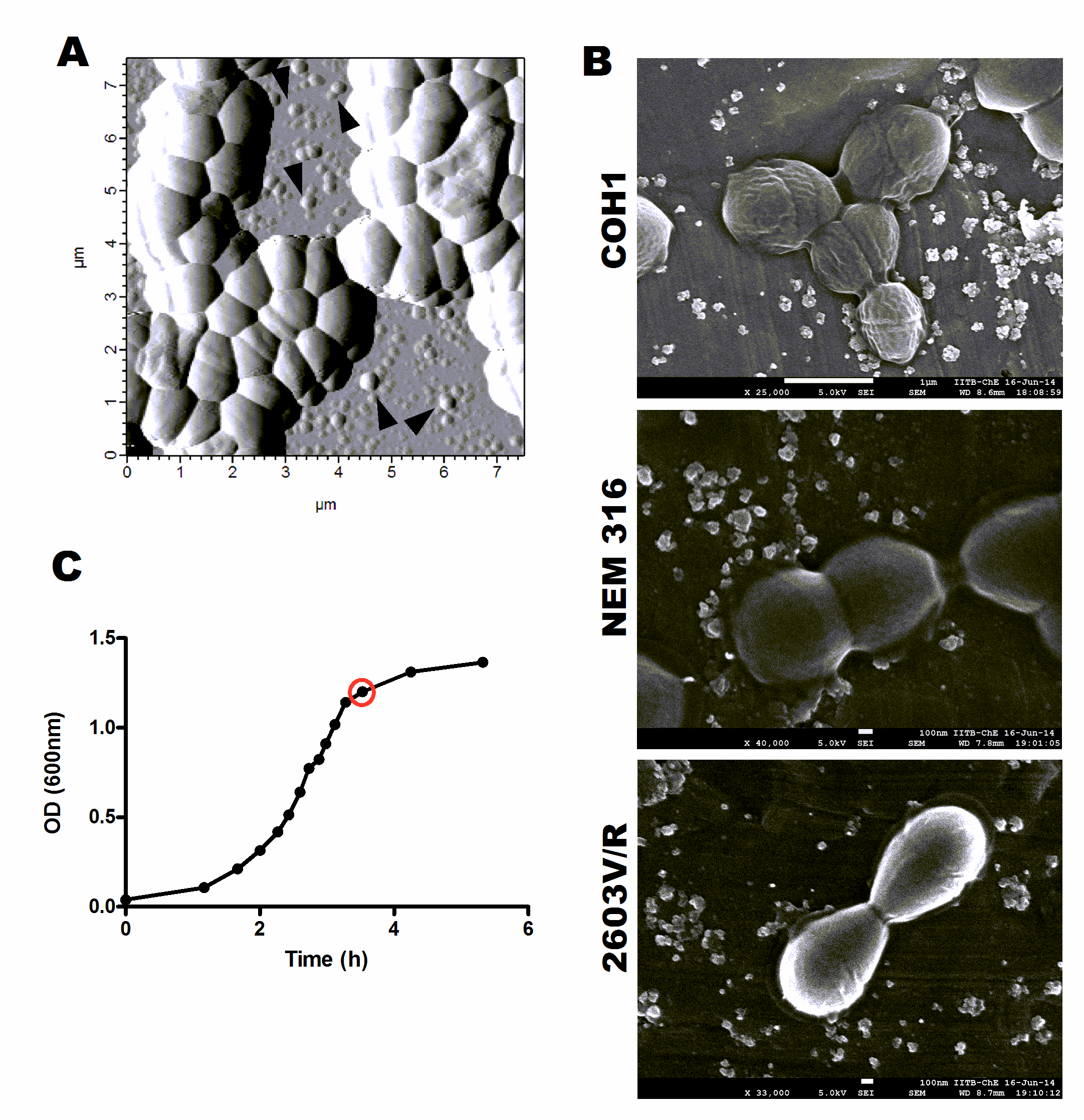

Supplement: S1 Fig — A. Atomic force micrograph of GBS strain A909 cells demonstrating presence of spherical MVs (black arrowheads) of different sizes around the cells. B. SEM analysis of different GBS serotype strains, COH1, serotype III; NEM316, serotype III; 2603V/R, serotype V; demonstrating secretion of MVs similar to serotype IA strain A909. Scale bar; 1 μm (for COH1) and 100 nm (for NEM316 and 2603V/R). C. Growth curve of GBS strain A909. Growth of bacteria was analyzed by measuring optical density at 600 nm (OD600) at different time intervals. OD value corresponding to 1.2 (late exponential phase of growth) has been marked with a red circle. (TIF) [file ppat.1005816.s001.tif]

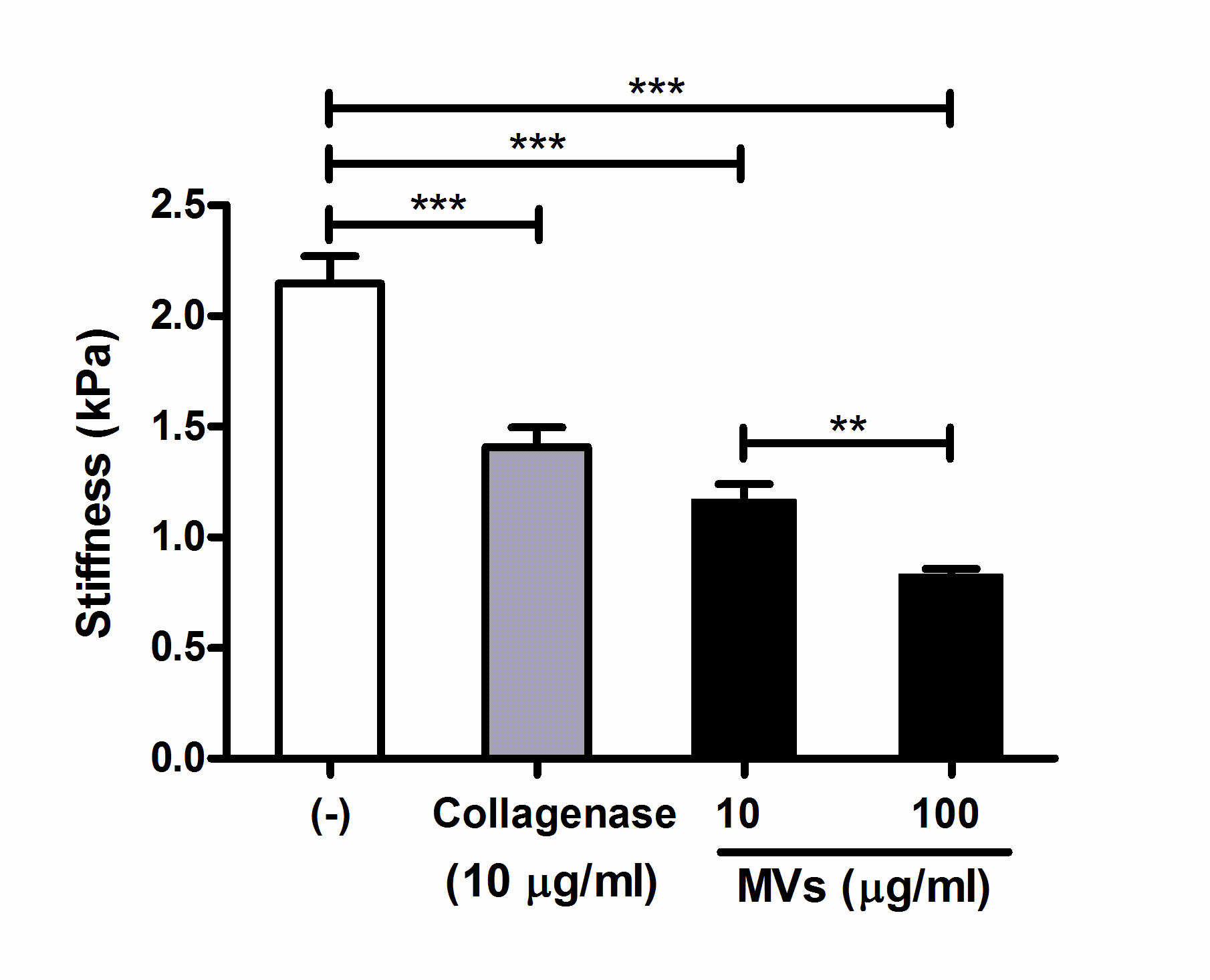

Supplement: S2 Fig — Mouse chorio-decidual membranes (on E14.5) were treated with either PBS, MVs or collagenase (10 μg/ml) for 16 h and probed at multiple positions over a randomly selected region of 50 μm X 50 μm to estimate average stiffness. Force-indentation curves were fitted with Hertz model to estimate the Young’s modulus of elasticity. Experiments were performed thrice and bars represent standard deviation of the mean of one representative experiment. Statistical analysis was performed using one-way ANOVA (Tukey’s multiple comparison test); ***p < 0.001; **p < 0.005. (TIF) [file ppat.1005816.s002.tif]

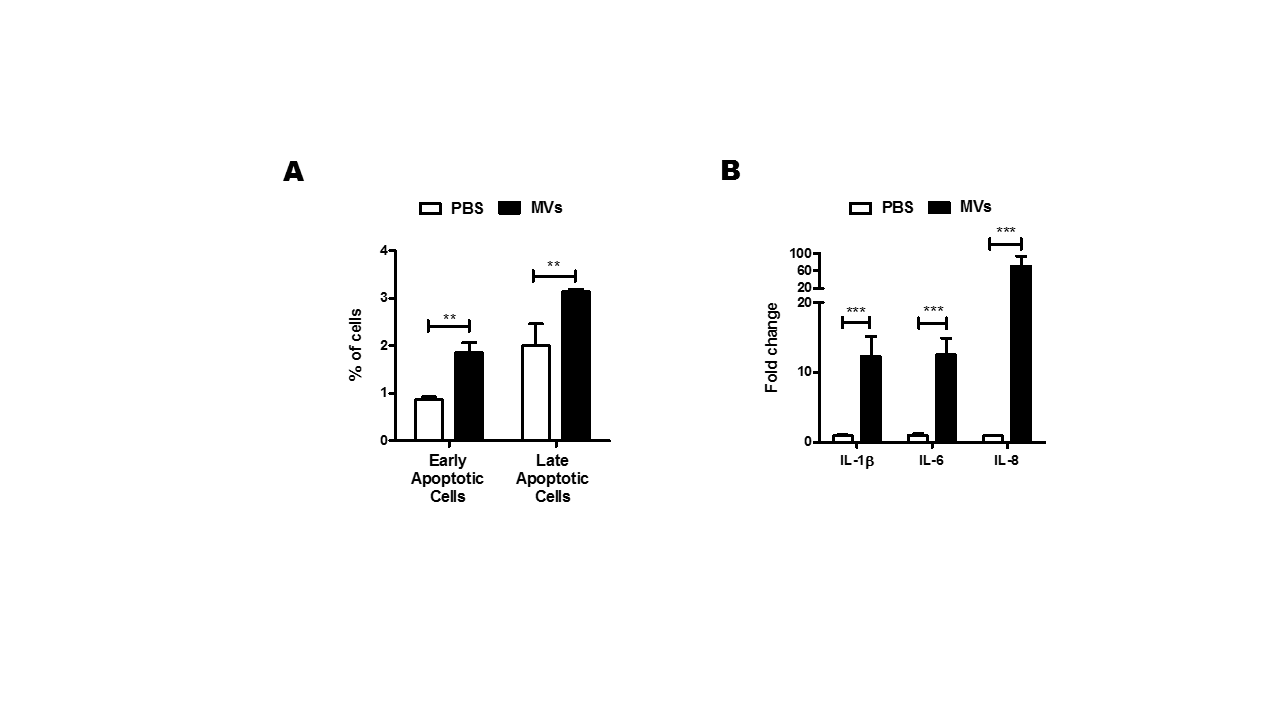

Supplement: S3 Fig — A. GBS MVs induced apoptosis in HeLa cells. HeLa cells were incubated with 100 μg/ml MVs for 24 h and stained with PI and/or Annexin V. Only Annexin V stained cells or PI and Annexin V double positive cells were analyzed by FACS and designated as early and late apoptotic cells, respectively. Experiments were performed thrice and bars represent standard deviation of the mean of one representative experiment. Statistical analysis was performed using one-way ANOVA (Tukey’s multiple comparison test); **p < 0.005. B. GBS MVs induced inflammatory response in HeLa cells. HeLa cells were incubated with 100 μg/ml MVs for 12 h and transcript levels of IL-1β, IL-6, IL-8 and β-actin were examined by qRT-PCR. Transcript levels were normalized to β-actin and expressed as fold change compared with cell treated with PBS only. Experiments were performed thrice and bars represent standard deviation of the mean of one representative experiment. Statistical analysis was performed using two-way ANOVA (Bonferroni test); ***p < 0.001. (TIF) [file ppat.1005816.s003.tif]

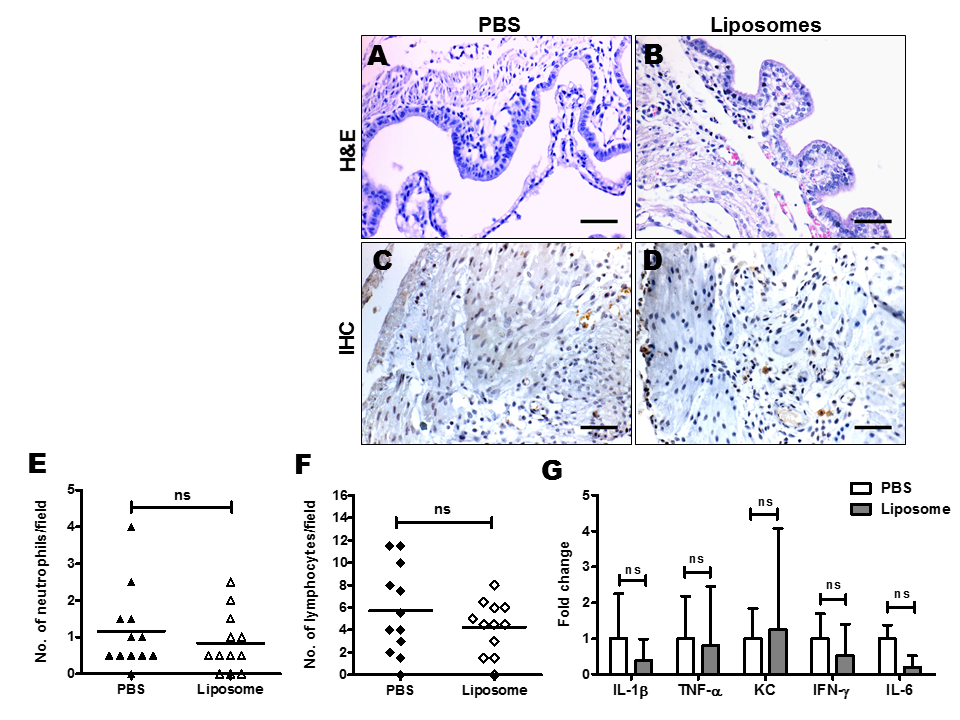

Supplement: S4 Fig — A-D. Intra-amniotic injections of PBS (A, C) or BSA encapsulated liposomes (B, D) were given to E14.5 day old pregnant mice and the embryos recovered on E15.5. Isolated chorio-decidua were fixed and stained with H&E as well as with anti-F4/80 Ab to detect presence of macrophages. Scale bar; 200 μm. E-F. Presence of neutrophils (E) and lymphocytes (F) were scored in different fields in chorio-decidual tissue that were injected with either PBS or liposomes. Statistical analysis was performed using Students t-test; ns, non-significant. G. mRNA levels of IL-1β, TNF-α, IFN-γ, KC and IL-6 was determined by qRT–PCR on total RNA isolated from mouse decidua, 24 h post injection with liposomes (n = 8). Transcript levels were normalized to 18s rRNA and expressed as fold change compared with tissues injected with PBS only. Statistical analysis was performed using two-way ANOVA (Bonferroni test); ns, non-significant. (TIF) [file ppat.1005816.s004.tif]
