# Supplementary material for: Membrane Vesicles of Group B Streptococcus Disrupt Feto-Maternal Barrier Leading to Preterm Birth
Source: PLoS Pathog. 2016 Sep 1;12(9):e1005816. doi: 10.1371/journal.ppat.1005816 (PMC5008812; doi:10.1371/journal.ppat.1005816)
Supplement: S1 Table — (DOCX) [file ppat.1005816.s005.docx]

**Suppl. Table 1. Oligonucleotide sequences.**

| **Primer Name** | **Oligonuleotide sequence (5´ to 3´ )** |
| --- | --- |
| CylE-F | CTGAAGCTTCCTTAGAAGATTATTCTGAAGTGGTTC |
| CylE-R | CTGTAATGCTCACATTTGTAGCATAAGGTGAGTT |
| Cfb-F | CATATGATGTATCTATCTGGAACTCTAGTGGCTGG |
| Cfb-R | GCTGTTTGAAGTGCTGCTTGTAATGTTACAATCTC |
| PepB-F | CTACTGTCTTTGCGACTGATGAGTTATGGG |
| PepB-R | GCT GCA TCC AGA TAG TCT GCA CTA GTC |
| ZooA-F | AGCGGATACTTATGTCCGTCCAA |
| ZooA-R | AGCAGTTCTTGACTGTTGTCAACGC |
| GapN-F | TATGTCAATGGCGAATGGAAATCATCTGTTAATCAG |
| GapN-R | GATACCTTGAACACCTGCACCAGATCC |
| IL1β-F | TGGAGAACACCACTTGTTGCTCCA |
| IL1β-R | AAACAGATGAAGTGCTCCTTCCAGG |
| IL6-F | GGAGACTTGCCTGGTGAAAA |
| IL6-R | CAGGGGTGGTTATTGCATCT |
| IL8-F | ATGACTTCCAAGCTGGCCGTG |
| IL8-R | GGAGTATGTCTTTATGCACTGACATCT |
| β-actin-F | GCTCGTCGTCGACAACGGCTC |
| β-actin-R | CAAACATGATCTGGGTCATCTTCTC |
| IL1β-F-mice | CTGATGAGAGCATCCAGCTTCA |
| IL1β-R-mice | CTTCTTTGGGTATTGCTTGGGATC |
| IL6-F-mice | TCCAGTTGCCTTCTTGGGAC |
| IL6-R-mice | GTACTCCAGAAGACCAGAGG |
| KC-F-mice | CCGCGCCTATCGCCAATG |
| KC-R-mice | CTTGGGGACACCTTTTAGCATCTTTTGG |
| IFNɤ-F-mice | GGCCATCAGCAACAACATAAGCGT |
| IFNɤ-R-mice | TGGGTTGTTGACCTCAAACTTGGC |
| TNFα-F-mice | TCTTCTCATTCCTGCTTGTGG |
| TNFα-R-mice | GGTCTGGGCCATAGAACTGA |
| 18s rRNA-F-mice | AACCCGGTGAGCTCCCTCCC |
| 18s rRNA-R-mice | TTCGAATGGGTCGTCGCCGC |
