# Supplementary material for: Membrane Vesicles of Group B Streptococcus Disrupt Feto-Maternal Barrier Leading to Preterm Birth
Source: PLoS Pathog. 2016 Sep 1;12(9):e1005816. doi: 10.1371/journal.ppat.1005816 (PMC5008812; doi:10.1371/journal.ppat.1005816)
Supplement: S2 Table — (DOCX) [file ppat.1005816.s006.docx]

|  | **Numbers of pups injected** | **Number of pups live born** | **IUFD (numbers)** | **Preterm (numbers)** | **Number of mice injected** |
| --- | --- | --- | --- | --- | --- |
| **PBS** | **31** | **27** | **1** | **3** | **4** |
| **5 µg** | **11** | **1** | **4** | **6** | **2** |
| **10 µg** | **34** | **1** | **10** | **23** | **4** |

**Suppl. Table 2. Fetal outcomes of intra amniotic injections with GBS MVs.**
